# Negative frequency-dependent selection through variations in seedling fitness due to genetic differentiation of parents’ pair in a tropical rainforest tree, *Rubroshorea curtisii* (Dipterocarpaceae)

**DOI:** 10.3389/fgene.2025.1552024

**Published:** 2025-02-06

**Authors:** Naoki Tani, Chin Hong Ng, Soon Leong Lee, Chai Ting Lee, Norwati Muhammad, Toshiaki Kondo, Yoshihiko Tsumura, Saori Sugiyama, Kaoru Niiyama, Azizi Ripin, Abdul Rahman Kassim, Samsudin Musa

**Affiliations:** ^1^ Forestry Division, Japan International Research Center for Agricultural Sciences (JIRCAS), Tsukuba, Japan; ^2^ Faculty of Life and Environmental Science, University of Tsukuba, Tsukuba, Japan; ^3^ Forestry Biotechnology Division, Forest Research Institute Malaysia (FRIM), Kuala Lumpur, Selangor Darul Ehsan, Malaysia; ^4^ Bioresource and Post-Harvest Division, Japan International Research Center for Agricultural Sciences (JIRCAS), Tsukuba, Japan; ^5^ Graduate School of Life and Environmental Science, University of Tsukuba, Tsukuba, Japan; ^6^ Department of Forest Vegetation, Forestry and Forest Products Research Institute, Forest Research and Management Organization, Tsukuba, Japan; ^7^ Advance Forest Resources, Rawang, Selangor Darul Ehsan, Malaysia; ^8^ Forestry and Environment Division, Forest Research Institute Malaysia (FRIM), Kuala Lumpur, Selangor Darul Ehsan, Malaysia

**Keywords:** dipterocarp, syngameon, genetic structure, fitness, frequency-dependent selection, tropical rainforest

## Abstract

**Introduction:**

The role of syngameons in adaption to microgeographical environmental heterogeneity is important and could be one of the sources of rich species diversity in tropical forests. In addition, negative frequency- or density-dependent selection is one of the major processes contributing to the maintenance of genetic diversity.

**Methods:**

To assess genetic factors that affect the fitness of seedlings of *Rubroshorea curtisii*, a dominant canopy tree species in hill dipterocarp forests, the inter- and intra-population genetic structure of individuals from natural populations and individuals at two permanent plots in a hill dipterocarp forest with reproductive stage was studied. Further, a total of 460 seedlings derived from six mother trees in the plot were raised in a nursery, and their pollen donors were identified using genetic marker based paternity assignment. Seed weight, bi-parental genetic relatedness, and bi-parental genetic heterogeneity based on the clustering analysis were used to analyze their effects on seedling fitness.

**Results:**

A Bayesian based clustering analysis revealed that three genetically distinct clusters were observed in almost all populations throughout the distributional range of the species in Malay Peninsula and provided the optimum explanation for the genetic structure of 182 mature individuals in the plots. The two clusters showed larger genetic differentiation from the ancestral admixture population, but the other one was not differentiated. The bi-parental larger genetic heterogeneity was associated with a significantly higher probability of seedling survivorship, and likewise, higher performance of vertical growth of the seedlings; but the seed weight and genetic relatedness did not significantly affect those.

**Discussion:**

This evidence suggests that fitter seedlings derived from mating between parents with different genetic clusters contribute to maintaining genetic diversity through negative frequency-dependent selection and may have an important role in adaptation in the tropical forest plant community.

## 1 Introduction

Stochastic and deterministic ecological mechanisms affect species co-existence in populations with high diversity and can allow the maintenance of a high diversity of species (Connell, 1961; Janzen, 1970; Hubbell, 1997; Webb and Peart, 1999; Wright, 2002; Usinowicz et al., 2017). The unified neutral theory of biodiversity and biogeography has provided an ecological null-model against which to test patterns of species diversity and composition within populations (Hubbell, 2001). However, species richness in tropical forests has been discussed as being the result of highly specialized adaptation to the biotic and physical environment, especially caused by forest disturbance (Ashton, 1969; Molino and Sabatier, 2001; Schnitzer and Carson, 2001; Alroy, 2017). Mechanisms to maintain species diversity in tropical forests have been studied; however, the adaptative potential of genetic diversity within tropical tree species and its effect on species diversity are poorly understood, despite the fact that genetic diversity is the basis of species diversity. It is frequently observed that tree species do not act as discrete evolutionary units (Cronk and Suarez-Gonzalez, 2018; Cannon and Petit, 2020). Closely-related and sympatric species often maintain some level of inter-specific fertility (Seehausen, 2004; Givnish, 2010; Schley et al., 2022), which can generate genotypes that facilitate further ecological diversification (the syngameon hypothesis; (reviewed in Seehausen, 2004). Therefore, the role of syngameons in adaption to microgeographical environmental heterogeneity is important and could be one of the sources of rich species diversity in tropical forests. In addition, negative frequency- or density-dependent selection is one of the major processes contributing to the maintenance of genetic diversity. At the species level, the high richness and co-existence of species in tropical forests has been partly maintained by density-dependent effects, appearing as reduced seed and seedling survival in areas with high densities or close proximity to conspecifics (the Jansen-Cornell hypothesis) (Janzen, 1970; Connell, 1971; Bagchi et al., 2010a; Bagchi et al., 2010b; Levi et al., 2019; Jia et al., 2020). This effect should also operate at syngameon and intra-species genetic diversity scales. Although negative-frequency dependent selection has theoretically been shown to be one of the mechanisms that maintains genetic diversity within populations, through the relative advantage of rare genotypes (Haldane, 1949; Ayala and Campbell, 1974; Antonovics, 1976), our understanding of the impact of frequency-dependent selection at syngameon and intra-species scales has focused on its maintenance of genetic and species diversity (Browne and Karubian, 2016; 2018; Eck et al., 2019; Cannon and Petit, 2020).

Tree species in the Dipterocarpaceae are highly diversified and form major components of the rainforest in Southeast Asia, in particular, the tribe Shoreae *sensu* Ashton (1979), which comprises five genera, *Dryobalanops*, *Hopea*, monotypic *Neobalanocarpus*, *Parashorea* and the large genus *Shorea* consisting of around 360 species. The genus *Shorea* has been traditionally classified based on timber characteristics (Symington, 1943) and *Rubroshorea* (= red meranti) is the largest among the six sections of *Shorea* (Maury-Lechon, 1978). Molecular phylogenetic approaches using various types of markers, such as PCR-RFLP (Tsumura et al., 1996; Indrioko et al., 2006) and plastid DNA sequences (Kajita et al., 1998; Dayanandan et al., 1999; Tsumura et al., 2011), have been used to examine these taxa. However, phylogenetic relationships have remained unclear, especially within the *Rubroshorea* due to low resolution of plastid DNA sequences (Tsumura et al., 2011). On the other hand, high-density genome wide DNA polymorphisms may be able to deliver high resolution of the species in the tribe Shoreae, similar to the traditional classification (Heckenhauer et al., 2018). Finally, *Rubroshorea* was proposed to dissected from genus *Shorea* based on molecular phylogenetic evidences (Ashton and Heckenhauer, 2022). Therefore, the highly diversified and closely related species belonging to *Rubroshorea* may be ideal models to study the evolutionary significance of syngameon complexes. *Rubroshorea curtisii* is commonly found in coastal and inland ridge forests throughout the Malay Peninsula (Symington, 1943) and is also confined to well-drained and fairly low nutrient soils along the northern coastal area in Borneo (Ashton et al., 1982). A low level of genetic differentiation between populations on the Malay Peninsula has been revealed by DNA polymorphisms in the chloroplast genome. However, chloroplast capture occurred through ancient hybridization events between *R. curtisii* and some unknown related species (Kamiya et al., 2012), and DNA sequences of two nuclear genes and the chloroplast have shown that hybridization occurs commonly between *R. curtisii* and *R. leprosula* and rarely between *R. curtisii* and *R. parvifolia* in natural populations (Kamiya et al., 2011). These species are morphologically distinct and, it has been suggested, are highly specifically adapted to their biotic and physical environments (Ashton, 1969; Brown and Whitmore, 1992; Potts et al., 2002; Dent and Burslem, 2016; Kenzo et al., 2019; Kenzo et al., 2023). Therefore, the genetic diversity and structure of *R. curtisii* are the result of stochastic processes, population history and hybridization events with extant (and ancient) closely related species.

We hypothesized that the multiple historical population and hybridization processes in *R. curtisii* caused the complex pattern of genetic diversity, which probably played an important role in adaption to the biotic and physical environments. To examine the hypothesis, the pattern of intra-specific genetic structure of *R. curtisii* in Malay Peninsula was assessed and the fitness of the seedlings categorized according to bi-parental genetic relationship and the pattern of genetic diversity. This approach can help in the understanding of the evolutionally and ecological importance of syngameons through detection of negative frequency dependent selection during mating and the subsequent regeneration of the forest ecosystem.

## 2 Materials and methods

### 2.1 Population sampling, seed collection and growth observation of seedlings

**FIGURE 1:**
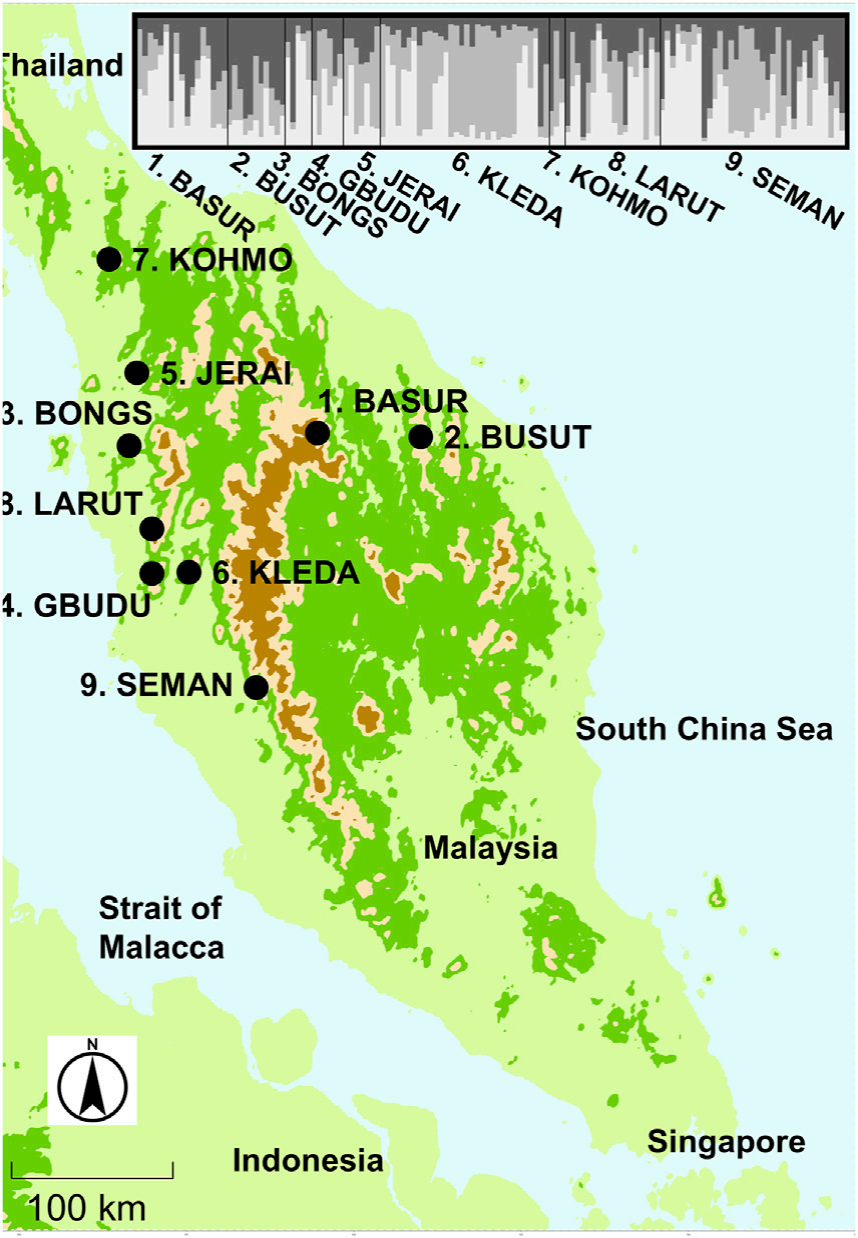
The locations of the sampled population of *Rubroshorea curtisii* trees on the Malay Peninsula and bar plot of *Q* values from STRUCTURE analysis for sampled individuals from nine populations.

Leaf or inner bark tissue was collected from 134 *R*. *curtisii* individuals, representing nine natural populations throughout the natural distribution across Malay Peninsula (Table 1; Figure 1). The tissues were collected from *R. curtisii* individuals that were at least 20 m apart, to avoid collecting samples from genetically related individuals, regardless of age or size of trees. Samples were stored at −20°C, prior to DNA extraction. Semangkok Forest Reserve, a designated hill dipterocarp forest conservation area, is in and governed by the Selangor state; it is 60 km north of Kuala Lumpur, on the Malay Peninsula and is a designated hill dipterocarp forest conservation area. In 1993, Niiyama et al. (1999) established a 6-ha permanent plot (200 m × 300 m) in an undisturbed forest on a narrow ridge and steep slope, ranging from 340 to 450 m above sea level (3°37′07″N, 101°44′15″E). Another ca. 4-ha (100 m × 400 m) permanent plot was established within a selectively logged area of forest in 1994 and was extended to about 5.4-ha (ca. 140 m × 400 m) in 2007 (3°37′23″N, 101°44′15″E); this is only 200 m away from the 6-ha permanent plot (Yagihashi et al., 2010). Leaf or inner bark samples were collected from 144 to 38 *R. curtisii* individuals (with dbh more than 20 cm) from the undisturbed plot and the logged plot, respectively, of which 17 and three trees were growing in areas of the study plots adjacent to the undisturbed and the logged plots, respectively. A total of 17 and three trees growing in areas of the study plots adjacent to the undisturbed plot have previously been identified as the candidate pollen donors (Tani et al., 2012; Tani et al., 2015). Samples were also stored at −20°C, prior to DNA extraction.

**TABLE 1:** Location and number of samples from the *Rubroshorea curtsii* population on Malay Peninsula.

No	Population name	Abbreviation	State	Sample size	Location	
					Latitude	Longitude
1	Gunung Basur	BASUR	Kelantan	17	N 05°38′02.8″	E 101°47′23.3″
2	Hulu Besut	BESUT	Trengganu	11	N 05°26′25.4″	E 102°25′21.9″
3	Gunung Bongs	BONGS	Kedah	5	N 05°20′45.4″	E 100°39′45.6″
4	Gunung Budu	GBUDU	Perak	6	N 04°39′19.8″	E 100°50′55.6″
5	Gunung Jerai	JERAI	Kedah	7	N 05°49′03.1″	E 100°40′27.4″
6	Kledang Saiong	KLEDA	Perak	32	N 04°32′48.7″	E 100°59′58.2″
7	Koh Moi	KOHMO	Kedah	3	N 06°26′04.0″	E 100°33′22.9″
8	Bukit Larut	LARUT	Perak	18	N 04°51′59.5″	E 100°46′28.5″
9	Semangkok	SEMAN	Selangor	35	N 03°38′31.9″	E 101°44′47.2″

A sporadic synchronized flowering event was observed in Semangkok Forest Reserve in October 2011, and fruit set occurred around February 2012. Seeds were collected from six selected mother trees in the undisturbed plot. After removal of wings from the seeds, seed weight was measured, then the seeds were placed on seedbeds consisting of river sand on 20 February 2012 to investigate germination. The result of the germination test is presented in Table 2. The germinated seedlings were potted in a mixture of river sand and soil without fertilizer on 26 March 2012 and maintained in the nursery of the Forest Research Institute Malaysia (FRIM, 3°14′01″N, 101°38′00″E) under 50% shade, with a water sprinkler system for irrigation. Height and survival of seedlings were monitored weekly before transplantation and monthly after transplantation. The data of seedling survival and growth were deposited in Dryad (https://doi.org/10.5061/dryad.nzs7h451m).

**TABLE 2:** Germination test for *Rubroshorea curtsii* seeds collected from six mother trees and result of paternity analysis for germinated seedlings with DNA retrievable.

				Paternity analysis				
Mother tree ID	Number of soaked seeds	Number of germinated seedlings	Number of seedlings with DNA	Selfing	Paternity donor unidentified (immigrants)	Multiple paternity donors identified	Single paternity donor identified	Number of paternity donors identified
E301	100	99	96	5	8	5	78	21
E311	99	78	71	3	12	4	52	14
F278	98	96	76	3	5	8	60	13
F324	100	79	60	11	4	5	40	19
G002	98	94	88	7	13	0	68	12
G108	93	77	69	12	6	4	47	15
Total	588	523	460	41	48	26	345	94

### 2.2 Molecular analysis

Genomic DNA was extracted, using the method described by Murray and Thompson (1980). The material analyzed came from inner bark tissue samples of the adult trees from the research plots, material from either the inner bark or leaf tissues for the population samples, and from the leaves of seedlings. The extracted DNA from the adult trees and population samples was further purified using a High Pure PCR Template Preparation Kit (Roche). After RNA digestion, the DNA was diluted to a concentration of about 2 ng/μL. All samples were genotyped on the basis of ten microsatellite markers (*shc04*, *shc07*, and *shc09* from Ujino et al. (1998), *sle074*, *sle384*, *sle392*, *sle562*, and *sle566* from Lee et al. (2004), and *slu044*, and *slu175* from Lee et al. (2006), see these references for details of microsatellite markers). Polymerase chain reaction (PCR) amplifications were carried out in total reaction volumes of 10 μL using a GeneAmp 9700 (Applied Biosystems). The PCR mixture contained 0.2 μM of each primer, 1x QIAGEN Multiplex PCR Master Mix (Qiagen), and 0.5–3 ng of template DNA. The temperature profile used was: 15 min at 95°C, then 30–35 cycles of 30 s at 94°C, 90 s at 50ºC–57°C and 90 s at 72°C, with a 10 min final extension step at 72°C. Amplified PCR fragments were electrophoretically separated using a 3100 genetic analyzer (Applied Biosystems) with a calibrated internal size standard (GeneScan ROX 400HD). The genotype of each individual was determined from the resulting electropherograms using GeneMarker (SoftGenetics). The microsatellite genotypic data from population samples, adult trees from the plots and seedlings from the nursery are referred as population genotype, plot genotype and seedling genotype, respectively. Due to fine-scale population structure and family structure in genotypes from adult trees and seedlings, quality of the microsatellite markers was checked using population sample in Semangkok forest reserve (abbreviated as SEMAN in Figure 1). No null alleles were detected in the Semangkok population, indicating a Hardy-Weinberg equilibrium state (Supplementary Table S1) by Micro-Checker analysis (Van Oosterhout et al., 2004). The plot genotypes were obtained in previous studies (Tani et al., 2012; Tani et al., 2015), and were already deposited in Dryad (http://dx.doi.org/10.5061/dryad.7k434). The population and seedling genotypes were separately deposited in Dryad (https://doi.org/10.5061/dryad.nzs7h451m).

### 2.3 Clustering analysis and paternity assignment

The Bayesian model-based clustering method implemented in STRUCTURE 2.3.4 (Pritchard et al., 2000) was used to estimate the optimal number of genetic clusters and probability of individuals belonging to each cluster (*k*) for population and adult data separately. A burn-in of 5 × 10^4^ steps followed by 10^5^ steps of MCMC (Markov chain Monte Carlo) simulations for both genotypes were performed using the admixture and allele frequency correlated model without the LocPrior option. Each analysis was run five times for the range *k* = 1 - 6 for both sets of data. In order to evaluate the likelihood of K, we uploaded the structure-generated results to a web-based program structure harvester (Earl and von Holdt, 2012) and obtained plots of mean likelihood value (Ln Pr*X*/*K*) and Delta *K* for successive values of *K*. We then determined the optimum values of *K*, following Evanno et al. (2005). For the selected *K* values, replicated results were aligned using CLUMPP version 1.1.2 (Jakobsson and Rosenberg, 2007), and visualized using distruct (Rosenberg, 2004) for population data.

We used categorical allocation in combination with an exclusion procedure to identify candidate paternal trees. The paternity of each offspring was determined based on likelihood ratios, and their confidence levels (greater than 95%) were derived using CERVUS ver. 3.0 (Marshall et al., 1998; Kalinowski et al., 2007). To conduct likelihood tests in CERVUS, we created 100,000 simulated offspring genotypes from 600 potential paternal candidates, with a mistyping rate of 0.1% in the categorical allocation. However, if the paternal candidates identified by the likelihood procedure had more than two loci mismatches in the simple exclusion procedure, we assumed that the paternal tree of the offspring was located outside the plot. Electropherograms were double-checked to confirm mismatches between the offspring and paternal candidates to minimize genotyping errors.

### 2.4 Survival and growth analysis

#### 2.4.1 Genetic relationship between parents of seedlings

We estimated the genetic relatedness (*r*_ij_) between the *i*th mother tree and the *j*th pollen donor within plots based on the microsatellite genotypes, using ML-Relate software (Kalinowski et al., 2006). Besides genetic relatedness, we also calculated two parameters to reflect the genetic heterogeneity between the parents of each seedling based on cluster analysis. As the probability of adult trees being assigned to the *k*th cluster was estimated as the *Q*_*k*_ value, the attributional difference of the STRUCTURE cluster between the *i*th mother tree and the *j*th pollen donor using *Q* values (referred to as the ‘*qdis*’ statistic) was calculated, which is an application of Rogers’ statistic for genetic distance between populations (Rogers, 1972), as follows,

$${qdis}_{ij}=\sum_{k=1}^{3}{\left(Q_{ik}-Q_{jk}\right)}^{2}/2$$

We also calculated another statistic whereby the attributional difference between the STRUCTURE cluster (*Q*_k_),and the genetic differentiation between the *k*th cluster and assumed ancestral admixed populations (*fst*_k_) were considered. The estimates of *fst*_k_ from STRUCTURE analysis were: 0.10552, 0.00044 and −0.10342 for 1^st^, 2^nd^ and 3^rd^ cluster (*k*th), respectively. This was used to calculate the so-called “*fdis*” statistic, calculated as follows,

$${fdis}_{ij}=\left|\sum_{k=1}^{3}\left(Q_{ik}+Q_{jk}\right)\times {fst}_{k}/2\right|$$

After these calculations, we assigned values of these statistics (*r*, *qdis* and *fdis*) to the parental pair of each seedling, based on the result of the paternity analysis.

#### 2.4.2 Survival analysis

In the seedling survival model, the status of the *s*th seedling at census time *t*, *N*_*s*_(*t*), was coded as 0 until the seedling was found dead, at which point it was set to *N*_*i*_(*t*) = 1 and no subsequent changes were considered. We used a proportional hazards model in the R statistical software using the package survival (Andersen and Gill, 1982; R Core Team, 2014; Therneau and Lumley, 2015) to fit maternal (seed weight represented as *w*) and biparental effects (*r*, *qdis* and *fdis*) to the survival of seedlings. As *qdis* and *fdis* were analyzed separately, we did not consider interaction between the explanatory variables. To select a best fit model among the combinations of the explanatory variables, and fixed or mixed models, we compared log-likelihood scores between candidate and null models and report the AICs of the models here.

#### 2.4.3 Seedling growth analysis

We analyzed the height-based relative growth rate for each seedling in response to the maternal (*w*) and bi-parental effects (*r*, *qdis* and *fdis*) using a generalized linear mixed model (GLMM) with normal distribution error structure. Although we monitored the seedling height growth every month, the vertical growth during each 6-month period was used for the estimation of relative growth rate (RGR) after transplantation into individual pots. RGR from *t*-1 to *t* observation dates for the *s*th surviving seedling was calculated as follows:

$${RGR}_{st}=\frac{\ln\left(\frac{H_{st}}{H_{st-1}}\right)}{T_{t}-T_{t-1}}$$

where *H*_*st*_ is the seedling height in centimeters at *T*_*t*_ observation date and *H*_*st-1*_ is the seedling height at *T*_*t-1*_ observation date (about 6 months before *T*_*t*_, the observation dates are shown in Table 4). A normal distribution was assumed for *RGR*_*st*_ for the GLMM. As the survival analysis, *qdis* and *fdis* were analyzed separately, no interaction between the explanatory variables was considered. Uninformative priors were given for the parameters’ initial distribution, then the distribution was re-parameterized with a Hamiltonian Monte Carlo sampler using the No-U-Turns algorithm. These models were implemented in stan 2.5 (Carpenter et al., 2017) using the package “Rstan” in R 3.1.1 (R Core Team, 2014; Gelman et al., 2015; Stan Development Team, 2020) and glmmstan function (Shimizu, 2016). Three chains were run for each survival and growth model with explanatory variables. All models were run for 150,000 iterations, discarding the first 30,000 as a burn-in period. We used the *Rhat* statistic, together with a visual inspection of the chains, to assess convergence (Gelman and Rubin, 1992). The effect of the explanatory variables on the growth of seedlings was evaluated according to whether the 95% posterior credibility intervals of the estimated coefficients included the value of zero.

## 3 Results

### 3.1 Genetic structure of *R. curtisii* at whole distribution and fine scales

The software structure applying a Bayesian estimate without prior population information produced the highest Delta *K* when the number of populations was set at three clusters (*K*_1 to 3_) for population genotype (Supplementary Table S2). We applied three clusters for the subsequent analyses. Although the genetic differentiation between the ancestral admixed population and clustered populations showed that two of the clusters were well differentiated (*F*_st1_ and *F*_st3_ were 0.16698 and 0.16244, respectively), the genetic component of the second cluster was similar to that of the admixed population (*F*_st2_ was 0.0006) when the population genotype was considered. On the other hand, the genetic differentiation between populations was lower (*F*_st_ = 0.082), which suggested that genetic components of the three clusters were distributed among most of the populations (Figure 1). This genetic clustering was also identified in adult tree populations in the plots. However, only the KLEDA population had a lower proportion for the *K*_2_ cluster and was like the genetic composition of the admixture.

**FIGURE 2:**
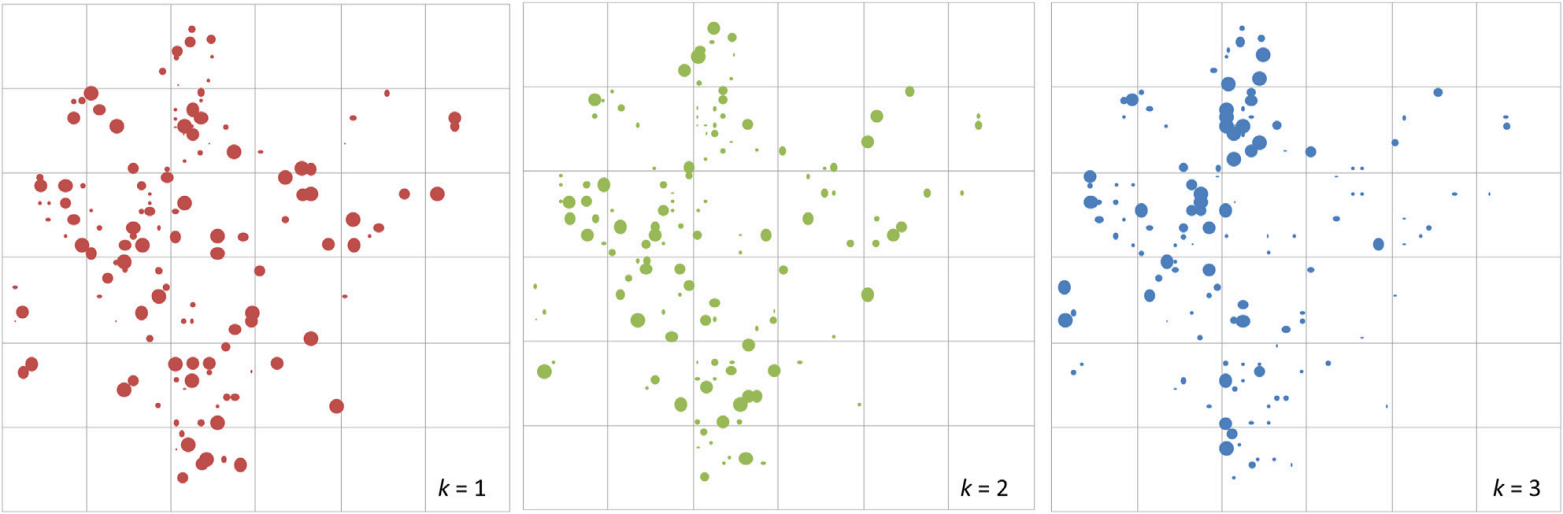
The distribution of adult trees of *Rubroshorea curtisii* (with dbh greater than 20 cm) in the 6-ha undisturbed plot at Semangkok Forest Reserve, Selangor, Malay Peninsula. The diameters of red, green and blue circles of the adult trees represent *Q* values for cluster 1, cluster 2 and cluster 3, respectively, from structure analysis.

At the fine scale, STRUCTURE analysis was also conducted using the plot genotypes in Semangkok Forest Reserve (the population genotypes of 9. Seman were collected from the vicinity of the forest monitoring plots, not within the plots). The adult trees in the plots also separated into three clusters at the fine scale. The fine-scale geographical distribution of *Q*_k_ showed some aggregation of large *Q*_k_ value individuals in all *k* clusters (Figure 2). Genetic differentiation between the ancestral admixed population and clustered populations showed the same tendency as the population genotypes, with *F*_stk_ values as follows, *F*_st1_: 0.10552, *F*_st2_: 0.00044, and *F*_st3_: 0.10552. These *F*_stk_ values were used for the calculation of *fdis*.

### 3.2 Germination, DNA collection and seedling paternity assignment

**FIGURE 3:**
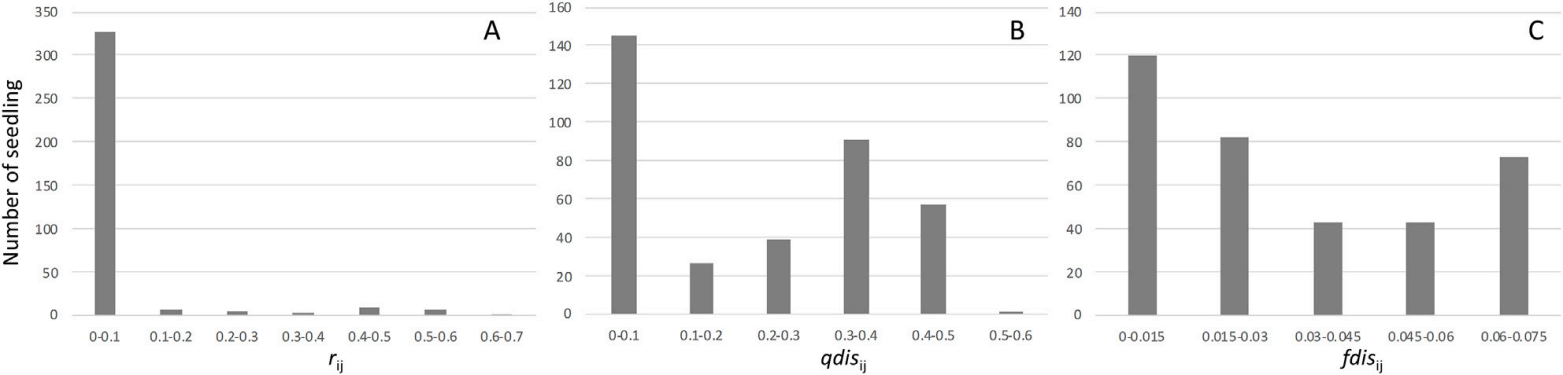
Frequencies of the genetic relationship between mother trees and pollen donors. Histogram **(A)** represents the distribution of relatedness between mother trees and pollen donors. Histogram **(B)** represents the distribution of *qdis* between mother trees and pollen donors. Histogram **(C)** represents the distribution of *fdis* between mother trees and pollen donors.

We collected nearly 100 seeds from each mother tree, which we soaked and placed on a seedbed of river sand on 30–31 January 2012. Seeds from three mother trees (E301, F278, and G002) showed almost 100% germination, while the other three mother trees (E311, F324, and G108) produced seeds with a lower germination rate, about 80%. After transplantation to pots, DNA was collected from leaf tissues of 60–96 individuals from each of the six mother trees, and this was used for paternity analysis (Table 2). Although the high exclusion power of the microsatellite markers was generally capable of assigning paternity to a single candidate or immigrant pollen from outside of the plot, 26 seedlings were excluded from subsequent analysis because there was no significant difference between the first and second candidate pollen donors. The paternity analysis showed 48 and 41 seedlings, respectively, derived from immigrant paternal donors and self-fertilization, and these were also excluded from subsequent analysis. As a result, 345 seedlings with single paternal donors inside the plots were used for further analysis (Table 2). Based on the paternity analysis, we evaluated the genetic relatedness between parents of the seedling (*r*), the attributional difference of clusters between the parents of seedling (*qdis*) and attributional difference of clusters with genetic differentiation (*F*_st_) between the parents of the seedling (*fdis*). The most of seedlings were the result of genetically unrelated mating between paternal donors and mother trees; therefore, the distribution of *r*_ij_ was skewed to the left (Figure 3A). However, the distribution of *qdis*_ij_ was almost normal, with the exception of many mating pairs between parents that belonged to distinct clusters (less than 0.1 *qdis*_ij_, Figure 3B). On the other hand, the effect of multiplying *F*_st_ changed the distribution of *fdis*. In particular, the difference in the probability of being assigned to the *k*_2_ cluster (*Q*_2_) between the parents of a seedling was masked by an almost zero value of *F*_st2_ and differences in the probability of being assigned to the *k*_1_ and *k*_3_ clusters were offset by subtraction of the difference for cluster *k*_1_ from cluster *k*_3_ (Figure 3C).

### 3.3 Survival, growth of seedlings of *R. curtisii* and the relationship with their genotype

Based on paternity assignment, maternal and biparental effects on seedling survival and growth were investigated using the proportional hazards model (“cox” in the R package) and GLMM using a Bayesian approach, respectively. For survival analysis, the *p* value of the estimated coefficient of *w* was slightly significant in Models 1 and 5, and the *p* value of the estimated coefficient of *r* was not significant in Models 2 and 5 in the “cox” analysis. However, the values of the *qdis* and *fdis* statistics consistently had a high level of significance throughout the models (Table 3). Among the models including *fdis* and *qdis*, the ones with *w* and *fdis* or *qdis* parameters showed the best prediction based on AIC and BIC (Models 6 and 7). In the comparison between *qdis* and *fdis*, the residual deviance of Model 7 (involving *w* and *qdis* parameters) was slightly smaller than that of Model 6 (involving *w* and *fdis*), but there was slight difference of 3.37 between them (Table 3). The biparental effect, represented by the attributional difference of clusters between the parents of seedling (*qdis*) and the attributional difference of clusters with genetic differentiation (*F*_st_) between the parents of the seedling (*fdis*), showed the best predictive ability to explain survival of *R. curtisii* seedlings in the nursery. The negative estimate of the *qdis* parameter represents the lower survival rate of seedlings with parents in the same cluster (based on the *Q* value in STRUCTURE). The positive estimate of the *fdis* parameter represents higher survival rates for seedlings resulting from mating between *k*_1_ and *k*_3_ type parents or mating between *k*_2_ type parents.

**TABLE 3:** Effects of seed weight, genetic biparental relatedness and difference of clustering attribution between parents on mortality of seedlings using COX proportional hazard model.

	Model 1	Model 2	Model 3	Model 4	Model 5	Model 6	Model 7	Model 8	Model 9	Model 10	Model 11
*w*	1.09*^a^				1.23**	0.74	0.79			0.81	0.85
	(0.44)^b^				(0.46)	(0.44)	(0.43)			(0.45)	(0.44)
*r*		0.91			1.36			0.27	0.19	0.57	0.51
		(0.80)			(−0.81)			(0.76)	(0.76)	(0.78)	(0.78)
*fdis*			21.77 ***			20.83 ***		21.48 ***		20.13 ***	
			(4.66)			(4.76)		(4.72)		(4.85)	
*qdis*				−3.4 ***			−3.28 ***		−3.37 ***		−3.2 ***
				(0.72)			(0.73)		(0.73)		(0.74)
df	2	2	2	2	3	3	3	3	3	4	4
AIC	1009.50	1014.1	992.75	989.91	1009.09	991.99	988.62	994.63	991.85	993.49	990.22
BIC	1012.06	1018.73	995.31	992.47	1014.13	997.04	993.66	999.67	996.89	1001.01	997.74
Deviance	1007.50	1012.11	990.75	987.91	1005.09	987.99	984.62	990.63	987.85	987.49	984.22

^a^ The number of astarisks represents significance level of estimated parameter, *, **, and *** indicate 5%, 1%, and 0.1%, respectively.

^b^ Number in pearenthesis is standard error of estimated parameter represented above.

The observation period (about 2 years and 8 months) during which seedling growth was measured was separated into the period of growth in the seedbed and five subsequent periods (each lasting 6 months) in the nursery after transplantation. The 95% credibility intervals of estimated parameters relating to seed weight and genetic relatedness between parents (*r*_ij_) included zero in all terms for both the *qdis* (Table 4) and *fdis* models (Table 5). However, the 95% credibility intervals of the estimated parameters for *qdis* were positively skewed from zero in the first and second 6-month periods in the nursery (Table 4). Those for *fdis* were negatively skewed from zero in the first and second 6-month periods in the nursery (Table 5). Any significance disappeared in the subsequent observation periods (Tables 4, 5). Only in the model involving *fdis*, was seed weight (*w*) positively correlated with seedling growth up to the third of the 6-month periods (Table 5), but the model involving *qdis* did not indicate any positive effect of seed weight (w) during any observation period (Table 4).

**TABLE 4:** Effects of attributional difference of clusters between the parents of seedling (*qdis*), relatedness (*r*) and seed weight (*w*) on relative vertical growth of seedlings in every 6 month period after transplantation.

	Mean	sd	2.5%	97.5%	Rhat
Vertical growth on seed bed (Feb/20/2012 - Mar/20/2012)					
Intercept	0.035	0.010	0.016	0.054	1.000
*qdis*	−0.005	0.013	−0.030	0.021	1.000
*r*	−0.010	0.019	−0.047	0.028	1.000
*w*	0.008	0.009	−0.010	0.026	1.000
sigma^a^	0.001	0.001	0.005	0.041	1.003
tau^b^	0.026	0.011	0.146	2.059	1.004
Vertical growth in the first 6-month period (Apr/17/2012 - Oct/23/2012)					
Intercept	0.003	0.000	0.002	0.004	1.136
*qdis*	0.001	0.001	0.000	0.002	1.071
*r*	0.001	0.001	0.000	0.002	1.009
*w*	−0.001	0.000	−0.001	0.000	1.110
sigma	0.000	0.000	0.000	0.000	1.213
tau	0.001	0.000	0.000	0.002	1.160
Vertical growth in the second 6-month period (Oct/23/2012 - Apr/8/2013)					
Intercept	0.004	0.000	0.003	0.005	1.002
*qdis*	0.002	0.001	0.001	0.003	1.004
*r*	0.000	0.001	−0.002	0.002	1.003
*w*	0.000	0.000	−0.001	0.001	1.004
sigma	0.000	0.000	0.000	0.000	1.012
tau	0.001	0.000	0.000	0.002	1.010
Vertical growth in the third 6-month period (Apr/8/2013 - Oct/23/2013)					
Intercept	0.002	0.000	0.001	0.003	1.002
*qdis*	−0.001	0.000	−0.001	0.000	1.004
*r*	−0.001	0.001	−0.002	0.000	1.000
*w*	0.000	0.000	−0.001	0.000	1.001
sigma	0.000	0.000	0.000	0.000	1.017
tau	0.001	0.000	0.000	0.001	1.011
Vertical growth in the fourth 6-month period (Oct/23/2013 - May/15/2014)					
Intercept	0.002	0.000	0.001	0.002	1.002
*qdis*	0.000	0.000	−0.001	0.000	1.046
*r*	−0.001	0.001	−0.002	0.000	1.011
*w*	−0.001	0.000	−0.002	0.000	1.002
sigma	0.000	0.000	0.000	0.000	1.127
tau	0.001	0.000	0.000	0.001	1.090
Vertical growth in the fifth 6-month period (May/15/2014 - Dec/24/2014)					
Intercept	0.000	0.000	0.000	0.001	1.001
*qdis*	0.000	0.000	−0.001	0.000	1.005
*r*	0.000	0.001	−0.001	0.002	1.000
*w*	0.000	0.000	0.000	0.001	1.002
sigma	0.000	0.000	0.000	0.000	1.008
tau	0.001	0.000	0.000	0.001	1.007

^a^ Sigma is a parameter of gaussian distribution of vertical growth.

^b^ Tau is a parameter of individual differences represented in random effect.

**TABLE 5:** Effects of attributional difference of clusters with genetic differentiation (*F*_st_) between the parents of the seedling (*fdis*), relatedness (*r*) and seed weight (*w*) on relative vertical growth of seedlings in every 6 month period after transplantation.

	Mean	sd	2.5%	97.5%	Rhat
Vertical growth on seed bed (Feb/20/2012 - Mar/20/2012)					
Intercept	1.751	0.441	0.879	2.590	1.003
*fdis*	−7.160	4.678	−16.213	2.206	1.001
*r*	−0.052	0.920	−1.868	1.760	1.000
*w*	2.669	0.473	1.745	3.604	1.002
sigma^a^	2.160	1.402	0.059	4.328	1.012
tau^b^	1.258	0.600	0.146	2.059	1.012
Vertical growth in the first 6-month period (Apr/17/2012 - Oct/23/2012)					
Intercept	4.628	0.968	2.706	6.523	1.000
*fdis*	−35.816	10.225	−55.932	−15.657	1.000
*r*	3.128	2.034	−0.833	7.134	1.000
*w*	2.974	1.046	0.919	5.029	1.000
sigma	9.859	6.839	0.234	20.317	1.003
tau	2.725	1.350	0.264	4.477	1.003
Vertical growth in the second 6-month period (Oct/23/2012 - Apr/8/2013)					
Intercept	11.382	2.089	7.298	15.548	1.000
*fdis*	−76.300	22.427	−119.675	−31.527	1.001
*r*	4.126	4.675	−5.013	13.183	1.001
*w*	7.172	2.255	2.676	11.596	1.001
sigma	42.489	33.436	0.558	90.406	1.031
tau	5.535	3.188	0.500	9.491	1.028
Vertical growth in the third 6-month period (Apr/8/2013 - Oct/23/2013)					
Intercept	6.543	1.801	3.044	10.101	1.001
*fdis*	−24.362	19.107	−61.071	13.423	1.001
*r*	−2.44	3.927	−10.035	5.266	1.001
*w*	3.81	1.962	−0.086	7.594	1.002
sigma	31.356	21.971	0.59	62.881	1.004
tau	4.499	2.524	0.442	7.844	1.005
Vertical growth in the fourth 6-month period (Oct/23/2013 - May/15/2014)					
Intercept	10.451	2.082	6.225	14.243	1.008
*fdis*	6.078	22.33	−38.528	47.322	1.012
*r*	−7.234	4.596	−15.837	2.173	1.006
*w*	−2.852	2.295	−7.035	1.841	1.007
sigma	33.336	28.316	0.224	77.084	1.012
tau	5.519	2.826	0.635	8.894	1.009
Vertical growth in the fifth 6-month period (May/15/2014 - Dec/24/2014)					
Intercept	1.703	2.467	−3.21	6.481	1.002
*fdis*	−4.873	26.497	−56.909	46.138	1.003
*r*	4.331	5.633	−6.872	15.269	1.006
*w*	4.789	2.71	−0.49	10.152	1.001
sigma	47.138	36.755	0.834	102.204	1.004
tau	5.983	3.275	0.608	10.072	1.005

^a^ Sigma is a parameter of gaussian distribution of vertical growth.

^b^ Tau is a parameter of individual differences represented in random effect.

## 4 Discussion

### 4.1 Population genetic structure of *R. curtisii*

The pattern of genetic structure of forest tree species has been shown to be related to only a few traits, such as mating system for the nuclear genome and seed dispersal mode or geographic range size for organelle markers (Duminil et al., 2007). The majority of dipterocarps adopt a mixed mating system (Kitamura et al., 1994; Lee et al., 2000c; Nagamitsu et al., 2001; Tani et al., 2009) and this is the case for *R. curtisii* (Obayashi et al., 2002; Tani et al., 2012; Tani et al., 2015). These species exhibit weak genetic differentiation between populations when there is no long-term geographic isolation, as is the case with other tree species in Malay Peninsula (Lee et al., 2000a; Lee et al., 2000b; Ng et al., 2017; Ng et al., 2019; Ng et al., 2024). On the other hand, comprehensive studies on the genetic structure of tropical tree species have suggested that limited seed dispersal enhances the genetic structure of populations (Hamrick et al., 1993; Lowe et al., 2018). Tropical tree species in Malay Peninsula with limited seed dispersal because of having wingless fruits also exhibit distinct genetic structure even though these species employ a mixed mating system (Ng et al., 2016; Lee et al., 2018; Ng C. H. et al., 2020). In the whole distributional scale, *R. curtisii* shows only two major distinct genetic clusters laying on northern major part and on southern tip of Malay Peninsula possibly due to the relatively larger genetic distinction at the southern populations (Ng et al., 2024). On the other hand, our analyzed population is within the range of main cluster of recent genetic diversity analysis. Although weak genetic differentiation was detected for the study species in Malay Peninsula, the *F*_*st*_ estimates of *k*_1_ and *k*_3_ in the Bayesian clustering analysis indicated large differentiation from the ancestral admixed population, which was the case in all studied populations except KLEDA (Figure 1), contributing to low genetic differentiation between populations. To date, there is no definitive evidence showing large genetic differentiation between the ancestral admixed population and the two clusters. However, a phylogenetic study using nucleotide variation of the chloroplast genome revealed that two distinct cpDNA haplogroups existed, differentiating the Malay peninsula and Borneo populations of *R. curtisii*, with several closely related species lying between the two lineages (Kamiya et al., 2012). Further, a nuclear *Pgi*C phylogeny showed that samples from Malaysia Peninsula and Borneo were clustered together and were distinct from other closely related species (Kamiya et al., 2005). The incongruence between cpDNA and nuclear DNA-based phylogenies may be caused by organelle capture during historical hybridization events with closely related species. Currently, natural hybridization events have been reported between *R. crtisii* and *R. leprosula* especially in the coastal distribution area of Malay Peninsula (Kenzo et al., 2019; Ng K. K. S. et al., 2020). Dipterocarp species may include large groups of species that exchange genes forming syngameon complexes or interfertile species (Cannon and Lerdau, 2015). The large differentiation of *k*_1_ and *k*_3_ clusters from the ancestral admixed population (which is close to the *k*_2_ cluster) may have possibility resulted from historical interspecific gene exchange between ancestral closely related species constituting a syngaemon. However, this would not be the result of current inter-specific hybridization between *R. curtisii* and *R. leprosula*, because the hybrids can be distinguished from the parent species by several leaf morphological characters, and some characters of the hybrids resemble *R. leprosula* more closely (Kamiya et al., 2012; Kenzo et al., 2019). None of our seedlings exhibited the intermediate type of leaf morphology (observation in nursery). On the other hand, a subspecies has been reported (*R. curtisii* Dyer ex King ssp. *grandis* P. S. Ashton), although this is rare and has been found only in two limited areas close to the KLEDA population. The wide distributional ranges of the three clusters, covering most of the studied populations, may indicate the maintenance of a syngaemon formed by ancestral genetic differentiation within species and among closely-related species. Further studies are required to gain an understanding of the population history and evolution of *R. curtisii*.

### 4.2 Seedling viability in relation to genetic composition of the parents

Two evolutionary episodes were assumed to explain the effect of the putative syngameon on seedling fitness. Seedlings with large *qdis* were the offspring of parents with very different *Q*_k_ estimates from each other. The *fdis* was designed to express the fact that the *k*_2_ cluster was intermediate between the *k*_1_ and *k*_3_ clusters. For example, the large *Q*_1_ value for *i*th mother tree of a seedling is neutralized by a large *Q*_3_ for *j*th paternal donor of the seedling when estimating *fdis*. In other words, a hybrid derived from mating between an individual with a high likelihood of belonging to the *k*_*1*_ cluster and another with a high likelihood of belonging to the *k*_*3*_ cluster is assumed to resemble the *k*_2_ cluster showing, with less genetic differentiation from the genetic make-up of the ancestral admixed population. The analyses to test the effects on survival showed that the absolute values of the *qdis* and *fdis* estimates are always larger than those of the other estimates, *r*, *w* (Table 3). Comparing *qdis* and *fdis*, deviances of models including *qdis* were always a little smaller than the corresponding models with *fdis*, indicating that the models with *qdis* explained survival of seedlings slightly better than those with *fdis*. Similarly, the early stages of growth were affected more by *qdis* and *fdis* than *r* and *w*, however, this tendency declined in the later stage of growth (Tables 4, 5). Thus, these variables indicating genetic differentiation between the parents of seedlings (*qdis* and *fdis*) can provide a sound explanation for the survival and initial growth of seedlings. This result helps us to understand the advantages of genetic differentiation, including the syngameon hypothesis, which may be linked to the maintenance of high tree species diversity in tropical rainforests. Seed dispersal limitation is typically associated with a spatially aggregated distribution pattern in tropical rainforest (Condit et al., 2000), which prevents tree species from recolonization and facilitates further aggregation of species complexes with intercrossing-ability (Cannon and Lerdau, 2015). The proximity of these mixed offspring could effectively allow them to back-cross, thus increasing mate choice with congeners and potentially re-establishing the local population (Baskett and Gomulkiewicz, 2011). This mechanism may enhance the fitness of seedlings from mother trees sired by pollen donors with different genetic backgrounds.

**FIGURE 4:**
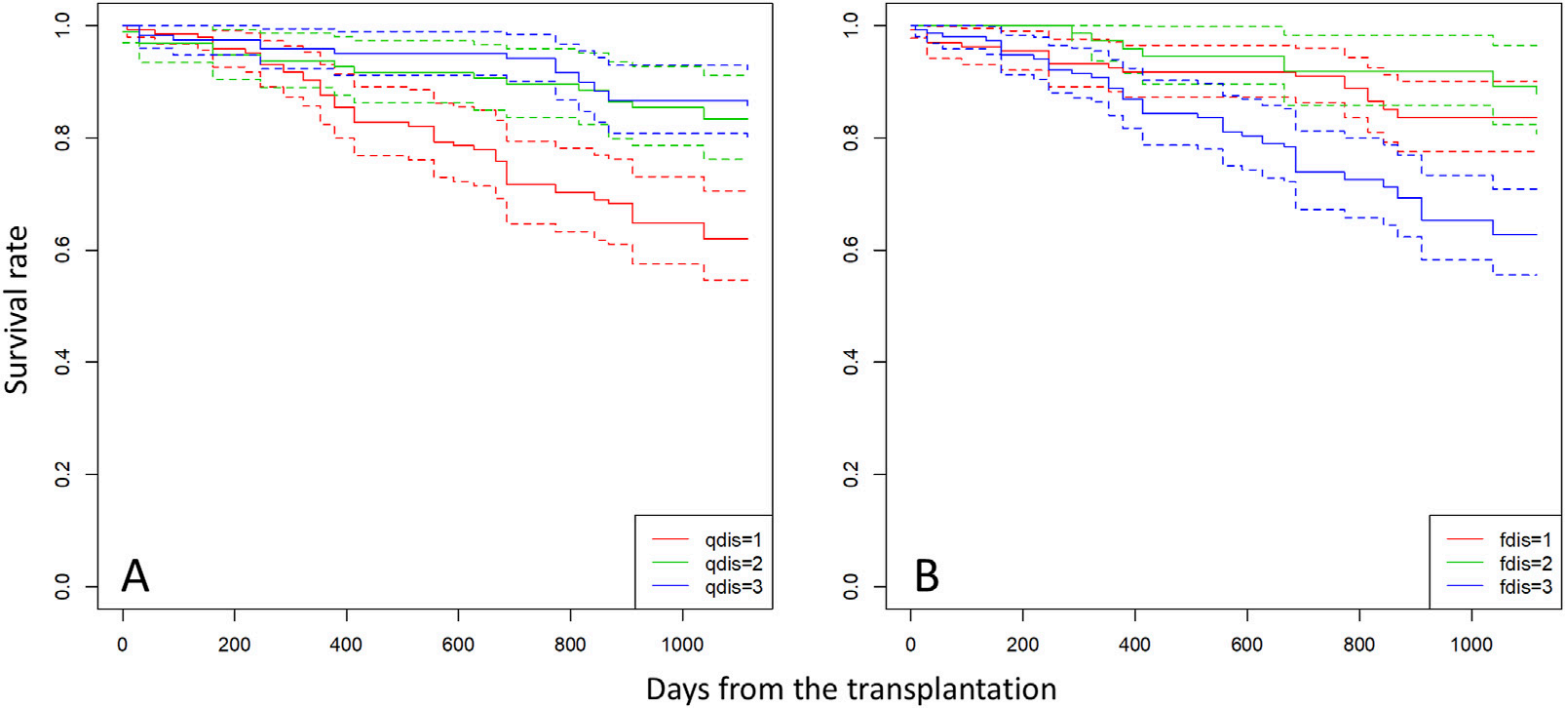
Survival curves for seedlings categorized into three groups based on *qdis* **(A)** and *fdis* **(B)** of each individual. Group “1” comprises seedlings with *qdis* < 0.1 and *fdis* < 0.02, group “2” comprises seedlings with 0.1 < *qdis* < 0.35 and 0.02 < *fdis* < 0.035, and group “3” comprises seedlings with *qdis* > 0.35 and *fdis* > 0.035.

In the mating and seedling establishment processes, inbred offspring (both selfing and biparental inbreeding) tend to be less successful due to inbreeding depression during fruit maturation (Tani et al., 2015) and the early stage of seedling establishment (Naito et al., 2005); therefore, *r* contributes less to survival and growth of seedlings in the subsequent stages of establishment. In the early stage of seedling development, ca. 40% of seedlings were derived from mating between parents with a similar probability of being assigned to clusters with less inbred mating (Figures 3A,B). These outcrossed seedlings produced by mating between parents with similar probabilities of being assigned to a particular cluster exhibited poorer survival throughout the observation period (Figure 4) and lower growth rates during the first year after transplantation (Table 4). This selection process can maintain genetic diversity through effects of overdominance and negative frequency-dependent selection (Ayala and Campbell, 1974; Asmussen and Basnayake, 1990). These effects have been noted as a mechanism to maintain genetic diversity in tropical forest tree species (Browne and Karubian, 2016; 2018). In natural stands, genetic diversity and the effects of overdominance and negative frequency-dependent selection may have positive effects with respect to escaping pathogens and herbivores of other trees. The Janzen-Connell hypothesis states that seeds or seedlings occurring at high densities or close to adult conspecifics are more vulnerable to attack from natural enemies such as pathogens and herbivores (Janzen, 1970; Connell, 1971). If a difference of compatibility between the enemies and genotypes exists, this may be another reason to maintain genetic diversity. Our seedings were grown in river sand in the seed bed and a mixture of river sand and organic soil in pots, produced by FRIM, so it was impossible to show that negative frequency dependent selection (represented by higher fitness of seedlings sired by a father with a different type of genetic diversity) protects offspring from enemies present on the mother trees. Therefore, it is very important that, in the future, we study survival and growth of seedlings in natural stands in relation to the genotypes of the seedlings and nearby adult trees.

We observed maintenance of strong genetic differentiation of the clusters in adult trees in the plots, the Semangkok population and at other levels (Figures 1, 2), which affected fitness of their seedlings. However, our seedling monitoring was conducted in a nursery, which can provide an ideal environment, including controlled light intensity and sufficient water. In natural stands, many dipterocarp species spend decades or more in the forest understory as seedlings (Delissio et al., 2002) and their ability to persist in the face of multiple stresses (e.g., pests, light, water and nutrient limitation) determines their chances of reaching the reproductive stage (Comita and Engelbrecht, 2014). Many studies have shown that drought is a major stress causing mortality of dipterocarp seedlings (e.g., Turner, 1990; Delissio and Primack, 2003; Bebber et al., 2004). Therefore, survival and growth of seedlings in relation to *qdis* and *fdis* statistics may represent different consequences under natural conditions. To understand the maintenance and evolutionary significance of the genetic differentiation pattern of clustering, the demographic analysis of natural seedlings with clear genetic structure is required. El Niño causes drought in Southeast Asian tropical forests and a greater possibility of drought is projected as a result of climate change in the near future (Amnuaylojaroen and Chanvichit, 2019; Rifai et al., 2019); this has the potential to cause failure of seedling recruitment and adversely affect seedling growth (Curran et al., 1999; Bebber et al., 2004; Condit et al., 2004; Itoh et al., 2012). Therefore, understanding adaptation of the species under various conditions, such as in managed and natural stands, is essential for conservation of tropical forests, especially in relation to climate change.

## Data Availability

The datasets presented in this study can be found in online repositories. The names of the repository/repositories and accession number(s) can be found in the article/Supplementary Material.
