# Supplementary material for: Negative frequency-dependent selection through variations in seedling fitness due to genetic differentiation of parents’ pair in a tropical rainforest tree, Rubroshorea curtisii (Dipterocarpaceae)
Source: Front Genet. 2025 Feb 6;16:1552024. doi: 10.3389/fgene.2025.1552024 (PMC11839620; doi:10.3389/fgene.2025.1552024)
Supplement: Supplementary file 1 [file Table1.docx]

| Supplemental Table 1. Table output of Micro-Checker using population dataset of SEMAN population | | | | | |
| --- | --- | --- | --- | --- | --- |
| **Locus** | **Null Present** | **Oosterhout** | **Chakraborty** | **Brookfield 1** | **Brookfield 2** |
| *shc04* | no | 0.0320 | 0.0273 | 0.0243 | 0.0243 |
| *shc07* | no | -0.0063 | -0.0039 | -0.0037 | 0.0000 |
| *shc09* | no | 0.0036 | 0.0080 | 0.0074 | 0.0074 |
| *sle074* | no | -0.0287 | -0.0304 | -0.0240 | 0.0000 |
| *sle384* | no | 0.0702 | 0.0685 | 0.0574 | 0.1236 |
| *sle392* | no | 0.0867 | 0.0721 | 0.0554 | 0.1832 |
| *sle562* | no | -0.0005 | 0.0012 | 0.0010 | 0.1299 |
| *sle566* | no | 0.0212 | 0.0146 | 0.0132 | 0.0132 |
| *slu044* | no | 0.0166 | 0.0031 | 0.0020 | 0.0020 |
| *slu175* | no | 0.0332 | 0.0332 | 0.0251 | 0.0251 |
| No loci show evidence for a null allele. | | | | | |
| This population is probably in Hardy Weinberg equilibrium. | | | | | |

Van Oosterhout, C., Hutchinson, W.F., Wills, D.P., and Shipley, P. (2004). MICRO‐CHECKER: software for identifying and correcting genotyping errors in microsatellite data. Molecular ecology notes 4(3), 535-538.

| Supplemental Table 2. Table output of the Evanno method (Evanno, Regnaut & Goudet (2005)) results from STRUCTURE HARVESTER (Earl and vonHoldt (2012)) for population genotype data | | | | | | |
| --- | --- | --- | --- | --- | --- | --- |
| ***K*** | **Reps** | **Mean LnP(*K*)** | **Stdev LnP(*K*)** | **Ln'(*K*)** | **\|Ln''(*K*)\|** | **Delta *K*** |
| 1 | 5 | -4917.62 | 2.152208 | — | — | — |
| 2 | 5 | -5054.64 | 69.47426 | -137.02 | 386.82 | 5.567817 |
| 3 | 5 | -4804.84 | 4.417352 | 249.80 | 244.80 | 55.41781 |
| 4 | 5 | -4799.84 | 7.324821 | 5.00 | 5.08 | 0.693532 |
| 5 | 5 | -4799.92 | 13.70518 | -0.08 | 20.6 | 1.503081 |
| 6 | 5 | -4820.6 | 16.20694 | -20.68 | — | — |

Evanno, G., Regnaut, S., & Goudet, J. (2005). Detecting the number of clusters of individuals using the software STRUCTURE: a simulation study. *Molecular Ecology*, 14(8), 2611-2620.

Earl, D. A., & von Holdt, B. M. (2012). STRUCTURE HARVESTER: a website and program for visualizing STRUCTURE output and implementing the Evanno method. *Conservation Genetics Resources*, 4(2), 359-361.
